# Endoscopic full-thickness dissection (EFTD) in the rectum: a case series

**DOI:** 10.1007/s10151-021-02558-w

**Published:** 2021-12-28

**Authors:** C. F. Rushfeldt, M. Nordbø, S. E. Steigen, T. Dehli, P. Gjessing, S. Norderval

**Affiliations:** 1 Department of Gastrointestinal Surgery, University Hospital of North-Norway, Tromsø, Norway; 2 Department of Pathology, Nordland Hospital, Bodø center, Bodø, Norway; 3 Department of Pathology, University Hospital of North-Norway, Tromsø, Norway; 4 Institute of Clinical Medicine, Faculty of Health Science, UiT, The Arctic University of Norway, Tromsø, Norway

**Keywords:** Rectum, Endoscopic full-thickness dissection, EFTD, Endoscopic full-thickness resection, EFTR, Rectal adenoma

## Abstract

**Background:**

Rectal endoscopic full- thickness dissection (EFTD) using a flexible colonoscope is an alternative to the well-established trans-anal endoscopic microsurgery (TEM) and the trans-anal minimally invasive surgery (TAMIS) techniques for resecting dysplastic or malignant rectal lesions. This study evaluated EFTD safety by analyzing outcomes of the first patients to undergo rectal EFTD at the University Hospital of North-Norway.

**Methods:**

The first 10 patients to undergo rectal EFTD at the University Hospital of North-Norway April, 2016 and January, 2021, were included in the study. The procedural indications for EFTD were therapeutic resection of non-lifting adenoma, T1 adenocarcinoma (AC), recurrent neuroendocrine tumor (NET) and re-excision of a T1-2 AC.

**Results:**

EFTD rectal specimen histopathology revealed three ACs, five adenomas with high-grade dysplasia (HGD), one NET and one benign lesion. Six procedures had negative lateral and vertical resection margins and in three cases lateral margins could not be evaluated due to piece-meal dissection or heat damaged tissue. Two patients experienced delayed post-procedural hemorrhage, one of whom also presented with a concurrent post-procedural infection. No serious complications occurred.

**Conclusion:**

Preliminary results from this introductory trial indicate that EFTD in the rectum can be conducted with satisfactory perioperative results and low risk of serious complications.

## Introduction

Rectal EFTD indications include non-lifting adenomas, T1 cancers with low risk of metastasis, and as palliative treatment for non-operable patients with T1 or T2 cancers.

Mesorectal fat circumferentially embeds the rectum below its peritoneal reflection. This enables endoscopic full-thickness resection of the rectal wall without the risk of leaking extra-luminal gas and bacterial fluid leading to collapse the intestinal lumen and contamination of the abdominal compartment.

Ovesco’s full-thickness resection device (FTRD) is a well-established method for excising colorectal lesions with good maneuverability; however, large lesion size and tissue rigidity provide this technique with significant limitations. Trans-anal endoscopic microsurgery (TEM) and the trans-anal minimally invasive surgery (TAMIS) are not limited by lesion size, but may be limited in their access to lesions located in the most proximal and distal rectum [1]. EFTD using a flexible colonoscope combines the maneuverability of the FTRD with the ability to resect larger lesions akin to that of the TEM and TAMIS techniques. EFTD is thereby effectively able to excise larger rectal lesions regardless of location resulting in a more versatile technique than FTDR, TEM or TAMIS.

Rectal EFTD has been employed for selected patients at the University Hospital of North-Norway since April 2016. This study retrospectively assesses the quality and the results of these procedures.

## Materials and methods

Between April 2016 and January 2021, ten patients with rectal lesions underwent EFTD at the Department of Gastrointestinal Surgery, University Hospital of North-Norway in Tromsø, Norway. These patients were prospectively registered in a database. A multidisciplinary team consisting of gastrointestinal surgeons, pathologists, radiologists and oncologists evaluated and determined patient suitability for excision of malignant rectal lesions using EFTD as well as evaluating further treatment, if cancer was found in the specimens after EFTD.

All patients received information regarding the procedure with an emphasis on potential complications including risk of delayed post-procedural hemorrhage and pelvic infection. Evaluation of rectal EFTD implementation as a replacement for TEM and TAMIS was categorized as a quality assurance project according to The Regional Committee for Medical and Health Research Ethics which did not require patient disclosure or consent.

All patients were lightly sedated with a customized combination of propofol and fentanyl. A gastrointestinal surgeon (C.R.) with extensive experience in therapeutic endoscopy performed all the procedures using an ultra-slim colonoscope (PCF-PH190I, Olympus, Hamburg, Germany). A submucosal lifting agent composed of a colloid solution (Voluven; Fresenius Kabi, Halden, Norway) (100 mL) and indigo carmine (2 mL) was used to separate the surrounding mucosa from the muscularis propria before both layers were circumferentially incised separately. A Triangle Tip knife (Olympus, Hamburg, Germany) and diathermic cutting forceps (SB-knife MD-47706, Medical device safety service, Hannover, Germany) were used for lesion incision and dissection as well as for hemostasis. A diathermic snare (SD-230U-20, Olympus) was used at the end of the procedure if the most central part of the stalk at the base of the lesion was difficult to access. Wall closure was performed with over-the-scope clips (14/6t, 100.14, Ovesco Endoscopy AG, Tübingen, Germany) and/or through-the-scope clips (Instinct Hemoclip, INSC-7-230-S, Cook Medical Europe, Limerick, Ireland). All patients received peri-procedural intravenous and post-procedural per oral prophylactic antibiotic treatments for 5 days.

Complications were registered in a database according to the Clavien–Dindo complication grading system [2]. Delayed post-procedural hemorrhage following rectal EFTD was defined as hemoglobin loss > 2 g/dL.

All patients had follow-up flexible rectal endoscopy. Follow-up was terminated prematurely due to advanced patient age in some cases.

## Results

In total, ten patients (six males and four females) were included in the study. Median patient age was 78 years (range 48–87) years. The overall technical success rate of rectal EFTD was 100% with a median procedure time of 119 min (range 65–191) minutes. Median post-procedural hospital length of stay was one night (range 0–3) nights.

Histological and therapeutic results are summarized in Table 1 with patients arranged in chronological procedural order. Indications for rectal EFTD included non-lifting adenoma in five cases, non-lifting recurrent adenoma in two cases, adenocarcinoma (AC) in two cases and recurrent neuroendocrine tumor (NET) in 1 case.

**Table 1:** Indications, morphology, pathological findings and clinical outcomes of EFTD cases

Patient / sex / age	Indication	Paris classification	Histology biopsies	Histology specimen	Resection margin	Complication (Clavien–Dindo)	Follow-up (months)	Status at follow-up
1/F/71	Non-lifting recurrent adenoma	IIc + IIa	LGD	HGD	Not assessable	No	14	No recurrence
2/M/48	Determine Sm-level of T1 AC	IIa	T1 AC	T1 AC, Sm3	R0	No	29	No recurrence^a^
3/F/73	Recurrent NET**	Submucosal	NET	NET	R0	No	10	No recurrence
4/M/82	Non-lifting adenoma Tumorhemorrhage	IIa	HGD	T2 AC	R0	Minor (II)	26	Recurrent rectal AC Suspicion of metastases
5/F/87	AC base resection Non-operable patient	Not relevant	T1-2 AC (resected specimen)	Benign (base of lesion)	Not relevant	No	17	Liver metastases
6/M/75	Non-lifting adenoma	IIa + Is	HGD	T1 AC, Sm1	R0	No	16	No recurrence
7/M/81	Non-lifting adenoma	IIa	HGD	HGD	R0	No	16	No recurrence
8/M/81	Non-lifting adenoma	IIa + Is	LGD	HGD	Not assessable	No	12	No recurrence
9/F/75	Non-lifting recurrent adenoma	IIc + IIa	LGD	HGD	R0	No	7	No recurrence
10/M/80	Non-lifting adenoma	IIa	HGD	HGD	Uncertain	Minor (IIIa)	3	No residual dysplasia

*EFTD* endoscopic full-thickness dissection, *LGD* low-grade dysplasia, *HGD *high-grade dysplasia, *AC* adenocarcinoma, *NET* neuroendocrine tumor

^a^Surgery after EFTD

The morphology of the eight superficial neoplastic lesions according to the Paris classification [3] is summarized in Table 1. Histological examination of pre-procedural biopsies revealed low-grade dysplasia (LGD) in three lesions, HGD in four lesions, AC in two lesions and NET in one lesion.

The median resected specimen size was 30 (9–35) mm in seven patients. Two resections were piecemeal and in one patient the size of the benign lesion was not recorded.

Histological examination of the rectal EFTD resected specimens showed HGD in 5 lesions, AC in 3 lesions, NET in 1 lesion and benign features in 1 lesion. Five lesions were histologically upgraded. The 3 ACs were classified as T1 Sm1, T1 Sm3 and T2. Patient 5 underwent primary endoscopic mucosal resection (EMR) of a T1-2 AC prior to EFTD. The full-thickness base of the excised lesion proved to be histologically benign. No synchronous lymph node metastasis or distant metastasis was discovered on peri-procedural computed tomography (CT) or magnetic resonance imaging (MRI) scans in patients with a malignant lesion.

Six rectal EFTD specimens were R0 resections, one was uncertain due to heat damage, one was benign and two were piecemeal resections without recurrence at follow-up.

Patients 4 and 10 experienced delayed post-procedural hemorrhage after 13 and 4 days, respectively. Patient 4 presented with a bleeding tumor prior to EFTD and was treated with blood transfusion following the procedure. Patient 10 was treated with endoscopic hemostasis. In both cases, bleeding occurred after resuming anticoagulant therapy. Patient 4 was also treated with antibiotics for a concurrent post-procedural infection. All complications were classified as minor (Clavien–Dindo II or IIIa, Table 1). No procedure-related mortality was observed.

Patients were followed up for a median of 13 (range 3–29) months with flexible rectoscopy and any lesion detected was biopsied. Recurrences were discovered in patient 4 and 5 after 26 and 17 months who underwent rectal EFTD for a T2 and a T1-2 AC, respectively.

**Fig. 1:**
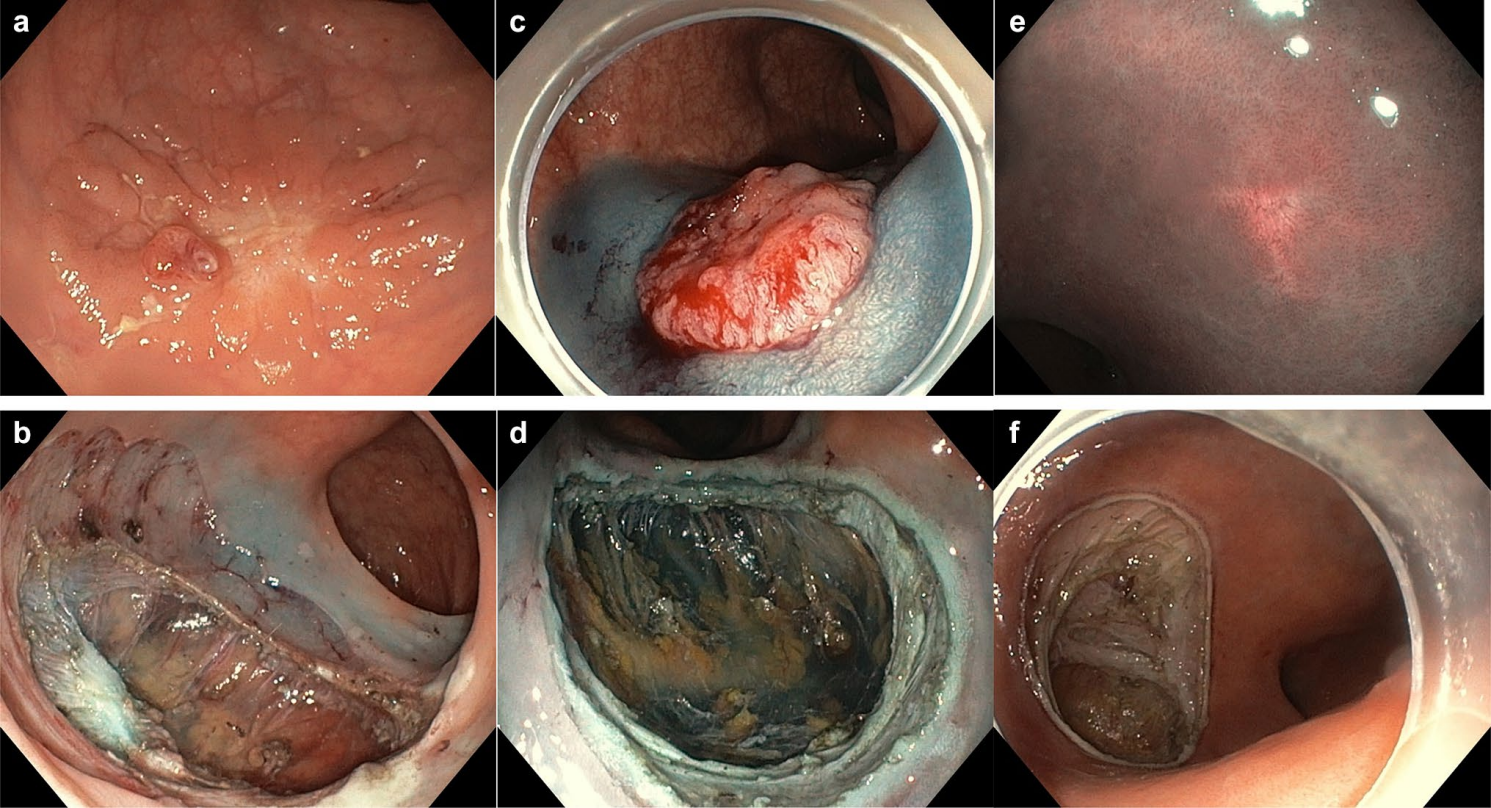
The rectal lesions of patients 1–3 prior to EFTD and the resultant full-thickness rectal wall defects. Patient 1: Recurrent adenoma with HGD before (**a**) and after (**b**) EFTD; Patient 2: T1 AC with blue lifting liquid before (**c**) and after (**d**) EFTD;Patient 3: a recurrent submucosal NET before (**e**) and after (**f**) EFTD. *HGD* high-grade dysplasia, *AC* adenocarcinoma, *EFTD* endoscopic full-thickness dissection, *NET* neuroendocrine tumor

**Fig. 2:**
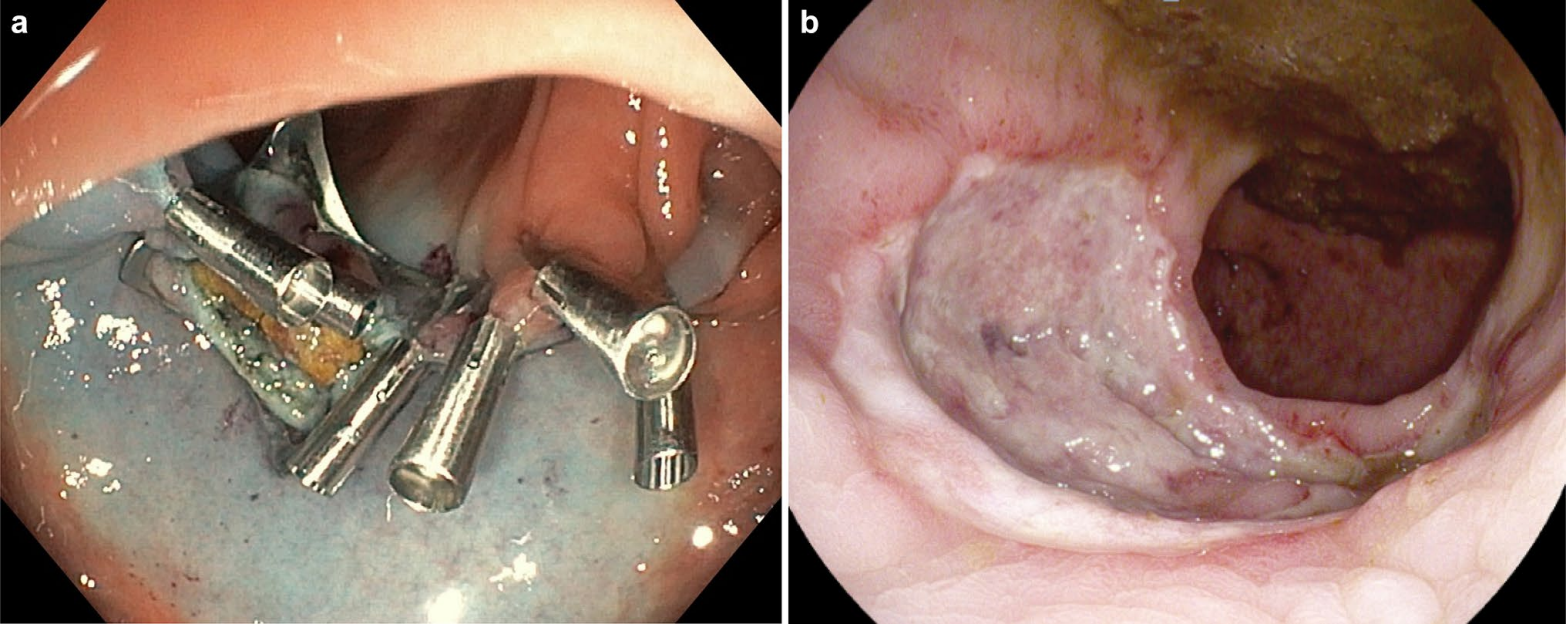
Closure of full-thickness defects. **a** Closure with one over-the-scope-clip and several through-the-scope clips (patient 2); **b** Open granulation 11 days after full-thickness resection (patient 10)

Figure 1 shows the rectal lesions of patients 1–3 prior to EFTD and the resultant full-thickness rectal wall defects. In patient 1 (Fig. 1a), a recurrent adenoma is resected piecemeal in two steps, including a primary EMR of the surrounding LGD adenoma, followed by a secondary EFTD of the non-lifting central HGD (Fig. 1b). In patient 2 (Fig. 1c), the mucosa surrounding a T1 AC is lifted with a blue lifting agent before the AC is fully excised (Fig. 1d). In patient 3, a recurrent submucosal NET (Fig. 1e) is resected (Fig. 1f). Figure 2 illustrates the closure of full-thickness defects with over- and through-the-scope clips in patient 2 (Fig. 2a) and by open granulation in patient 10 (Fig. 2b).

## Discussion

This study reports a series of the first ten patients to undergo EFTD of rectal lesions using a flexible colonoscope at our institution. To our knowledge, this is the first reported series apart from 1 case report [4] in the PubMed literature detailing EFTD of rectal lesions.

Procedural indications for EFTD included non-lifting lesions in seven cases, a recurrent submucosal NET in one case, base resection of a previously resected T1-2 AC in one case and diagnostic resection of a T1 AC (patient 2) in one case. While endoscopic submucosal dissection (ESD) is a technique suitable only for lifting lesions, EFTD is indicated for non-lifting lesions due to submucosal fibrosis, invasive growth or tumors originating from the muscularis propria. In this study, ESD could therefore only have been implemented in the case of patient 2. In a patient unsuitable for rectal surgery with a T1 AC biopsy, it is debatable whether ESD or EFTD should be performed to ensure sufficient radicality, as preoperative investigations with regard to early T-stage invasion depth are uncertain [5].

Trans-anal full-thickness resection of rectal lesions has been performed for several years using the TEM and TAMIS techniques, which employ rigid laparoscopic instruments and a metal anoscope or rubber anal port, respectively. The large diameter of the anal port and anoscope is necessary for introduction of the laparoscopic instruments into the rectal cavity and effective maneuvering therein, but for TAMIS, it may in turn hinder access to lesions in the very distal rectal ampulla close to the anal canal [6]. Furthermore, due to the rigidity of the laparoscopic instruments, lesions in the most oral part of the rectum may be difficult to access due to anatomical deviation of the rectum [1]. With a flexible endoscope, both the aforementioned areas of the rectum are accessible, which make the colonoscope a more versatile tool than the rigid laparoscopic instruments of the TEM and TAMIS techniques. The size and efficiency of the laparoscopic instruments cannot be compensated for by the smaller endoscopic instruments of the colonoscope. Therefore, use of the TEM and TAMIS instruments is likely less time consuming for larger rectal lesions. On the other hand, use of the TEM and TAMIS techniques routinely requires patient admission to hospital, spinal or general anesthesia [7] and a more stringent patient arrangement to ensure the rectal lesion is at the 6 o’clock position. All EFTD procedures in this study were performed under light sedation and in a fixed lithotomy position. For all of the above reasons, EFTD has replaced TEM and TAMIS for the resection of benign and premalignant rectal lesions in our department.

Despite a median patient age of 78 years, all EFTD procedures in this study were initially performed in an outpatient setting. However, five patients were converted from observation to inpatient status for one night, and one patient was admitted for three nights. These were in large part due to comorbidity and/or logistical issues such as being operated toward the end of the day with long transportation route home.

Ovesco’s FTRD equipment is well established as an all-inclusive full-thickness device that closes wall defects after resection. It is cited as the most commonly used technique for full-thickness resections of rectal and colonic lesions [8] and is almost synonymous with endoscopic full-thickness resection (EFTR) in published literature [8, 9]. This report aims to highlight the clear distinction between Ovesco’s FTRD technique and the EFTD technique used in this case series. While the small diameter and cap volume of the FTRD technique prevent it from being effective in resecting larger or more rigid lesions, the EFTD technique does not possess these limitations.

Resection margins were evaluated and reported in all nine lesions where the pathologist encountered any degree of dysplastic cells and/or malignancy, and is therefore omitted in one case where the re-excision yielded a benign specimen (patient 5). Of the nine reported cases six (67%) were R0, two were not assessable due to piecemeal resection and one was uncertain due to heat damaged tissue. All three latter cases had tumor free resection margins macroscopically and more importantly no recurrences or residual dysplasia was seen at follow-up.

In toto, EFTD of neoplastic lesions allows the pathologist to evaluate radicality of the excised lesion, which has traditionally necessitated a rectal wall defect larger than the diameter of the lesion. Non-lifting adenomas often have good peripheral lift but poor lift centrally where invasion initially occurs in most cases. If one defines R0 resection only in regard to the invasive part of the lesion, then the surrounding adenoma can quickly and efficiently be removed by endoscopic mucosal resection (EMR) or ESD followed by EFTD of the centrally invasive part. This limits the size of the wall defect while safely removing the invasive component of the lesion in its entirety. This approach was used in patients 1 and 8 (Table 1, Fig. 1a) with no sign of recurrences at follow-up at 14 and 12 months, respectively. This technique is also described for non-lifting adenomas in the colon using the Ovesco FTRD device combined with EMR [10]. Further studies are needed to evaluate the efficacy of this approach with respect to recurrence.

The relatively long median procedure time of 2 h should be assessed in light of the innovative nature of this new technique. We expect operating time to decrease relative to the individual operator experience gained in future procedures. Perhaps the greatest advantage of EFTD, and one which may save time overall, is the possibility of rapid conversion from EMR/ESD to EFTD if the central area of a rectal adenoma does not lift.

Patients 4 and 10 experienced post-procedural hemorrhage after 13 days and 4 days, respectively. Patient 4 also experienced a concomitant infection. In patient 4, the rectal wall defect was closed following EFTD, and in patient 10, the wall defect was not closed. Both cases occurred after resuming anticoagulant therapy. In future, anticoagulant therapy will only be resumed 2 weeks following rectal EFTD if other indications do not necessitate earlier resumption.

The infection in patient 4 was detected by elevated C-reactive protein levels without fever or pelvic pain. A CT scan could not exclude a pelvic infection due to air present in and around the mesorectal fat tissue. The infection was successfully treated by replacing routine per oral postoperative antibiotics with intravenous broad-spectrum antibiotics.

Because mesorectal fat tissue circumferentially envelopes the wall of the rectum below its peritoneal reflection, even large full-thickness defects will normally heal without complications. The main disadvantage with intraoperative wall closure is a prolonged operating time. With the TEM and TAMIS techniques, full-thickness defects in the rectum may be closed endoscopically with standard laparoscopic instruments and a low-cost suture provided that the defect is not too large. Suturing after EFTD is also possible with an OverStitch-equipped flexible colonoscope but at a much higher cost [11]. In this case series 6 of 10 full-thickness defects were closed with over-the-scope clips and/or through-the-scope clips and 4 healed by open granulation. In a multicenter randomized controlled trial comparing rectal wall defects closed versus those not closed following TEM, there was no significant difference between the two groups with respect to postoperative infection, hemorrhage or pain on days 1 and 7 [12].

The mesorectal fascia must be kept intact during EFTD to avoid seeding tumor cells into the pelvic region, thereby thwarting any later completion surgery. In patients with AC in preoperative biopsies and expected low T stage, EFTD should primarily be reserved for those unfit for major rectal resections, and as a palliative procedure, albeit with the possibility of a curative outcome.

Patients with AC in preoperative biopsies and/or in EFTD resection specimens were evaluated for further treatment by a multidisciplinary team based on lesion histology, patient age, comorbidity and metastatic status. Patient 6, who had a T1 Sm1 AC, was not recommended rectal resection due to advanced age and low risk of metachronous metastasis [13]. Biopsy at 16 months revealed no recurrence. Patient 2, who had a T1 Sm3 AC, was offered a low anterior rectal resection after EFTD due to high risk of metastasis, young age and lack of comorbidity. Histopathological examination of the resected rectum revealed no lymph node metastasis or malignancy. Patients 4 and 5, who had a T2 and T1-2 AC, respectively, were not candidates for rectal resections due to advanced age and severe comorbidities. Patient 5 presented with radiologically suspected liver metastasis at 17 months and died only a few days thereafter. No liver or rectum biopsies were performed. Patient 4 presented with a local recurrence, a radiologically suspected mesorectal lymph node metastasis and a solitary liver metastasis after 26 months. Rectal resection was still not an alternative due to the aforementioned reasons. Palliative radiation therapy was attempted but not tolerated, and the patient died 2 months following diagnosis of the recurrence.

In the setting of the non-operable patient, as in the case of patients 4 and 5, it is debatable whether radiation therapy or local resection of a T2 rectal AC is the best option. Radiation therapy was not indicated for patient 4 as biopsies only contained HGD. Similarly, radiation therapy was not indicated for patient 5 as malignancy was not recognized before EMR of T1-2 AC (supplemented with an EFTD of the tumor base). Further, adjuvant radiation therapy is not indicated following a R0 resection with no suspicion of lymph node metastasis.

This study shows that two elderly and comorbid patients received rectal EFTD treatment for a T1-2 and T2 AC, which provided the patients 17 and 26 disease-free months without recurrence. EFTD may therefore serve as an alternative to radiation therapy in a palliative and life-prolonging setting. Finally, this case series suggests that EFTD of rectal AC less invasive than T2 (patient 6) can yield satisfactory surgical radicality without dysplastic or malignant recurrences within the limited follow-up period of this study.

The main limitations of this study are the low number of patients included as well as the lack of randomized controlled trials including the aforementioned established techniques for resecting rectal dysplastic and malignant lesions.

## Conclusion

This study demonstrates that rectal EFTD may represent an alternative to the TEM, TAMIS and Ovesco FTRD techniques in situations where these techniques are insufficient. Further studies focusing on the risk of recurrence are needed to clarify the long-term safety of excising dysplastic or malignant rectal lesions using EFTD.

## Data Availability

Data are available in the electronic patient journals as well as in a protected Excel file.
